# Photocatalytic H_2_ Evolution Using Different Commercial TiO_2_ Catalysts Deposited with Finely Size-Tailored Au Nanoparticles: Critical Dependence on Au Particle Size

**DOI:** 10.3390/ma7127615

**Published:** 2014-11-26

**Authors:** Ákos Kmetykó, Károly Mogyorósi, Péter Pusztai, Teodora Radu, Zoltán Kónya, András Dombi, Klára Hernádi

**Affiliations:** 1 Research Group of Environmental Chemistry, Institute of Chemistry, University of Szeged, Tisza L. krt. 103., H-6720 Szeged, Hungary; E-Mails: kmetykoakos@chem.u-szeged.hu (A.K.); k.mogyorosi@chem.u-szeged.hu (K.M.); hernadi@chem.u-szeged.hu (K.H.); 2 Department of Applied and Environmental Chemistry, University of Szeged, Rerrich tér 1., H-6720 Szeged, Hungary; E-Mails: peter.pusztay@gmail.com (P.P.); konya@chem.u-szeged.hu (Z.K.); 3 Faculty of Physics, Babeș-Bolyai University, M. Kogălniceanu 1, RO-400084 Cluj-Napoca, Romania; E-Mail: teocluj@gmail.com

**Keywords:** TiO_2_, H_2_ evolution, Au nanoparticle, size-dependent activity, oxalic acid

## Abstract

One weight percent of differently sized Au nanoparticles were deposited on two commercially available TiO_2_ photocatalysts: Aeroxide P25 and Kronos Vlp7000. The primary objective was to investigate the influence of the noble metal particle size and the deposition method on the photocatalytic activity. The developed synthesis method involves a simple approach for the preparation of finely-tuned Au particles through variation of the concentration of the stabilizing agent. Au was deposited on the TiO_2_ surface by photo- or chemical reduction, using trisodium citrate as a size-tailoring agent. The Au-TiO_2_ composites were synthetized by *in situ* reduction or by mixing the titania suspension with a previously prepared gold sol. The H_2_ production activities of the samples were studied in aqueous TiO_2_ suspensions irradiated with near-UV light in the absence of dissolved O_2_, with oxalic acid or methanol as the sacrificial agent. The H_2_ evolution rates proved to be strongly dependent on Au particle size: the highest H_2_ production rate was achieved when the Au particles measured ~6 nm.

## 1. Introduction

There is currently an increasing demand for clean energy sources; harvesting sunlight has a huge potential in this area. Solar energy can be converted and stored indirectly as chemical energy by producing H_2_ through heterogeneous photocatalysis [1,2]. As TiO_2_ can be excited within the near-UV spectral range, the development of a suitable TiO_2_-based photocatalyst could be beneficial for this procedure. For efficient H_2_ production in an irradiated TiO_2_ suspension, there are two main prerequisites: (i) efficient hole scavenging by an organic compound that may be readily oxidized in the absence of molecular O_2_, and (ii) the presence of active surfaces without an overvoltage for H_2_ formation.

The overvoltage of H_2_ evolution can be decreased by modifying the TiO_2_ surface with noble metals (mostly Au [3,4,5,6], Pd [7], Pt [8,9,10], and Ag [11]). Such surface metal nanoparticles can also decrease the electron-hole recombination rate, leading to a better photocatalytic performance [12,13]. The most common procedure for noble metal deposition is impregnation of the TiO_2_ surface with a noble metal ion-containing solution, followed by drying. The noble metal ions are reduced either before or after the impregnation. Post-impregnation reduction can be carried out, for example, by heating the sample in a H_2_ flow [14,15] or with formaldehyde [16]. Photoreduction is also an appropriate process for the precipitation of noble metal nanoparticles onto the catalyst surface: TiO_2_ is excited by UV irradiation, and a suitable organic compound in the reaction mixture is therefore oxidized, while the noble metal nanoparticles are reduced. Examples of such sacrificial organic compounds as good hole scavengers are methanol [17,18], oxalic acid [13,19], and 2-propanol [20]. Photoreduction can also be performed without the presence of any organic compound, but it is then slower or needs irradiation at higher energies [21,22]. Another possibility for the generation of noble metal nanoparticles is the addition of a reducing agent to the solution containing the noble metal precursor, e.g. hydrazine [23,24], ascorbic acid [25], citrate [26], or sodium borohydride [27,28,29]. The effectiveness of the catalyst depends strongly on the amount of noble metal loaded onto the TiO_2_ surface. The active sites of the catalyst can be blocked if there are too many metal nanoparticles on the surface, while if the metal content is too low, the desired activity enhancement might not be achieved.

H_2_ can be generated photocatalytically from pure water, but water is only a moderate hole scavenger. The H_2_ evolution rate can be elevated several-fold if the reaction mixture contains readily oxidizable organic compounds. Azo dyes [30], different alcohols [31,32,33,34,35,36], chloroacetic acid [37], oxalic acid [19], formic acid [38], acetic acid [21], glucose [39], glycerol [40], *etc.* might be suitable hole scavengers. Many of these organic compounds commonly occur as byproducts in industrial wastewaters. It would be a cost-effective and environmentally friendly solution if such wastewaters could be purified through the use of solar radiation and H_2_ molecules were produced at the same time.

It has been demonstrated that the catalytic activity in different catalytic processes may be strongly size-dependent [41]. However, there have been few publications concerning the size dependence for photocatalytic hydrogen production. Gold nanoparticles were grown in different sizes on a titania surface by Murdoch and co-workers and photocatalytically tested [42]. However, the number of samples in the 1–10 nm range was very limited and the gold content in the samples also varied.

Our present aim was therefore to find an easily adjustable synthesis method with good reproducibility to reduce Au nanoparticles with controlled size onto the TiO_2_ surface at constant Au content. The main objective was an extensive comparison of the H_2_ evolution rates on these photocatalysts as a function of the noble metal particle size in organic compound-containing suspensions.

## 2. Experimental

### 2.1. Catalyst Preparation

Two commercially available TiO_2_ powders were used as bare catalysts: Aeroxide P25 (average particle diameter 25.4 nm, 90% anatase + 10% rutile) and Kronos Vlp7000 (average particle diameter 7.8 nm, 100% anatase). All the syntheses and photocatalytic tests were carried out in Millipore Milli-Q ultrapure water as medium. Gold nanoparticles were deposited on the TiO_2_ surface by photoreduction (PR) or chemical reduction (CR) methods.

#### 2.1.1. Photoreduction Procedure

The UV photoreduction of Au(III) ions is accelerated if the reaction mixture contains a hole-scavenging organic compound. Either oxalic acid (OA; Scharlau, extra pure) or trisodium citrate (TC; ≥99.0%, Sigma-Aldrich Co., St. Louis, MO, USA) was used as hole-scavenger (samples PROA and PRTC, respectively).

The total volume of the reaction mixture was 35 mL. The calculated amount of TiO_2_ was suspended in water (5 g/L), and HAuCl_4_ × 4H_2_O (Reanal, analytical grade) was added to achieve a concentration of 2.5 × 10^−4^ M, followed by the hole-scavenging organic compound (*c*_OA_ = 5.0 × 10^−2^ M or *c*_TC_ = 2.5 × 10^−4^ M). The suspension was next subjected to UV irradiation to allow photoreduction of the noble metal. Within 1–5 min, there was a characteristic color change from white to dark purple, which indicated the formation of Au nanoparticles. After irradiation for 1 h, the suspension was washed by centrifugation in the presence of oxalic acid (5.0 × 10^−2^ M) to improve the sedimentation and to eliminate the remaining Cl^−^ and Na^+^. The final suspension was used fresh for photocatalytic tests without any further processing.

#### 2.1.2. Chemical Reduction Procedure

In this procedure, different concentrations of trisodium citrate (2.50 × 10^−4^ M, 1.88 × 10^−4^ M, 1.25 × 10^−4^ M and 0.63 × 10^−4^ M) were used to stabilize the forming Au nanoparticles and to grow Au nanoparticles of different sizes. The reaction mixture was thermostated at 20 °C. TC was added to the TiO_2_ suspension (*c*_TiO2_ = 5 g/L), followed by HAuCl_4_ (*c*_HAuCl4,final_ = 2.5 × 10^−4^ M). Finally, freshly-prepared, ice-cold NaBH_4_ (Aldrich, purum) solution was added as a reducing agent (*c*_NaBH4, final_ = 3 × 10^−3^ M). The suspension immediately turned purple. As the reduction took place in the presence of TiO_2_, this procedure was designated CRIS (chemical reduction, *in situ*). After a 1-h wait, the suspension was washed by centrifugation as described in Section 2.1.1. The redispersed catalyst was used immediately for the photocatalytic experiments.

Other Au-doped TiO_2_ catalysts were prepared by mixing the Au sol with the TiO_2_ suspension after the chemical reduction (chemically reduced sol-impregnated samples, CRSIM). The washing procedure was the same as for the CRIS Au-TiO_2_.

**Figure 1:**
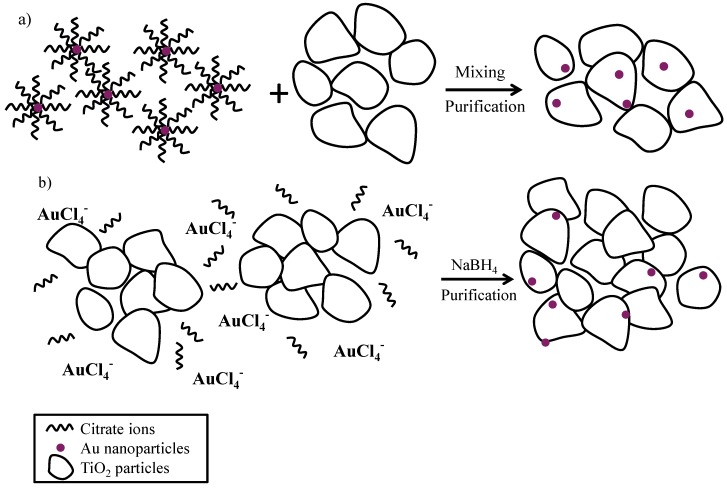
Synthesis of Au-TiO_2_ samples by chemical reduction: (**a**) Chemically reduced sol-impregnated sample (CRSIM); (**b**) chemical reduction *in situ* synthesis (CRIS).

The CRIS and CRSIM procedures are outlined schematically in Figure 1. The main reason for the use of these two experimental routes was to find optimum conditions for the deposition of gold. Citrate anions can help stabilize the positively-charged Au nanoparticles in the step of nucleation growth, providing size-focused and nearly monodisperse noble metal nanoparticles [27,43,44].

### 2.2. Characterization of the Catalysts

#### 2.2.1. Spectrophotometry

The UV-VIS spectra of the Au sols were measured in 1 cm quartz cells in an Agilent 8453 diode array spectrophotometer (Agilent Technologies, Santa Clara, CA, USA), with Millipore MilliQ ultrapure water as blank.

#### 2.2.2. Transmission Electron Microscopy (TEM)

The average size of the Au nanoparticles deposited on the Aeroxide P25 TiO_2_ was calculated from TEM images recorded with a 100 kV Phillips CM 10 instrument (FEI, Hillsboro, OR, USA), using formvar-coated Cu grids.

#### 2.2.3. X-ray Diffraction (XRD)

It was difficult to differentiate the Au nanoparticles from the Kronos Vlp7000 TiO_2_ particles of almost the same size in TEM images. For these samples, therefore, the average Au particle diameter was determined from the line broadening of the XRD peak of Au at 38.2° (2θ), using the Scherrer equation.

XRD measurements were performed on a Rigaku diffractometer (CuK_α_ = 0.15406 nm, 30 kV, and 15 mA, in the regime 35° ≤ 2θ ≤ 42° for solid powder samples (Rigaku Co., Kent, UK).

#### 2.2.4. Energy-Dispersive X-ray Spectroscopy (EDX)

To determine the exact Au loading on the prepared Au-TiO_2_ samples, we used EDX. SEM–EDX analysis was performed on a Hitachi S-4700 Type II cold field-emission scanning electron microscope attached to a Röntec QX2-EDS spectrometer (Röntec AG, Berlin, Germany). No conductive coating was applied on the samples.

#### 2.2.5. BET Specific Surface Area

To investigate the influence of noble metal loading on the surface area of the catalysts, the BET method was used. The specific surface areas of the catalysts were determined via the adsorption of N_2_ at 77 K, using a Micromeritics gas adsorption analyzer (Gemini Type 2375, Micromeritics, Aachen, Germany).

#### 2.2.6. X-ray Photoelectron Spectroscopy (XPS)

XPS measurements were performed on a SPECS PHOIBOS 150 MCD instrument (SPECS GmbH, Berlin, Germany), with monochromatized Al Kα radiation (1486.69 eV) at 14 kV and 20 mA, and a pressure lower than 10^−9^ mbar. Samples were mounted on the sample holder through the use of double-sided adhesive carbon tape. High-resolution Au4f, Ti2p and O1s spectra were recorded in steps of 0.05 eV for the analyzed samples. The data obtained were analyzed with CasaXPS software.

### 2.3. H_2_ Production Measurements

The freshly-prepared, washed catalyst was suspended in 50 mM oxalic acid solution and poured into a glass reactor (total volume: 150 mL), irradiated by 10 × 15W UV fluorescent lamps (λ_max_ = 365 nm, LightTech Kft., Budapest, Hungary). The well-stirred suspension (*c*_catalyst_ = 1 g/L) was purged with N_2_ (99.995%, Messer Kft., Budapest, Hungary) at a flow rate of 50 mL/min to ensure O_2_-free conditions. The reactor was connected through a PTFE tube to a Hewlett Packard 5890 gas chromatograph fitted with a 5Å molecular sieve column and a thermal conductivity detector. Samples were taken from the gas flow with a 2 mL sampling valve, every 10 min in the first hour of the experiment and every 20 min in the second hour. The rate of H_2_ evolution was calculated with regard to the GC calibration (carried out with certified 5% H_2_:N_2_ gas) and the N_2_ flow rate.

### 2.4. UV Decomposition of Oxalic Acid

These experiments were carried out under the same conditions as in the H_2_ production measurements, but liquid samples were taken from the suspensions at intervals during the reaction, and the residual oxalic acid and total organic carbon (TOC) concentrations were measured. Following centrifugation and filtration with a Whatman Anotop 25 0.02 μm syringe filter, the HPLC measurements were performed on a Merck Hitachi device fitted with an L-4250 UV-VIS detector (Merck KGaA, Darmstadt, Germany) and a GROM Resin ZH 8 μm column. The TOC contents of the samples were measured in suspensions with an Analytik Jena multi N/C 3100 instrument.

## 3. Results and Discussion

### 3.1. Size of Au Nanoparticles

#### 3.1.1. Spectra of Au Solutions

**Figure 2:**
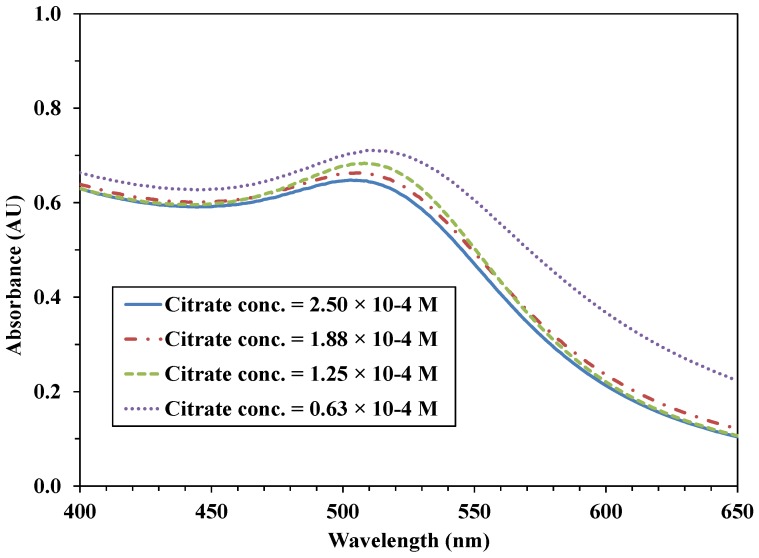
Absorption spectra of Au sols synthesized with different TC concentrations.

The UV-VIS spectra of the nanoparticles synthesized by the CRSIM method displayed a plasmon peak at around 510 nm, characteristic for Au nanoparticles, causing the red-purple color of the solutions. With decreasing initial TC concentration, the band broadened and a red shift was observed (Figure 2). This means that larger particles were formed because of the lower initial concentration of the stabilizing agent. It should be mentioned that with both procedures, the supernatants of the suspensions containing the Au-TiO_2_ samples were all colorless, which indicates that the Au nanoparticles were all well stabilized on the TiO_2_ surface.

#### 3.1.2. Particle Size of Au on TiO_2_

**Figure 3:**
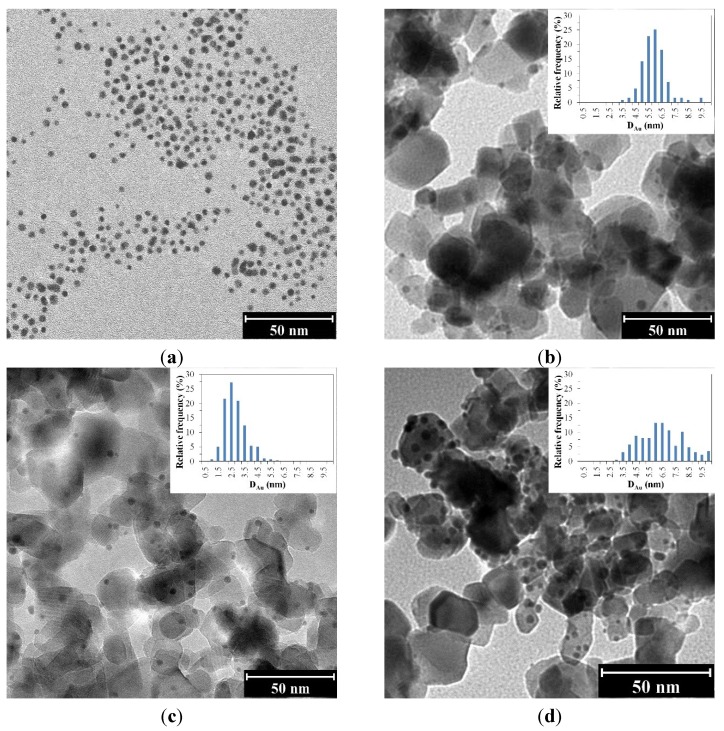

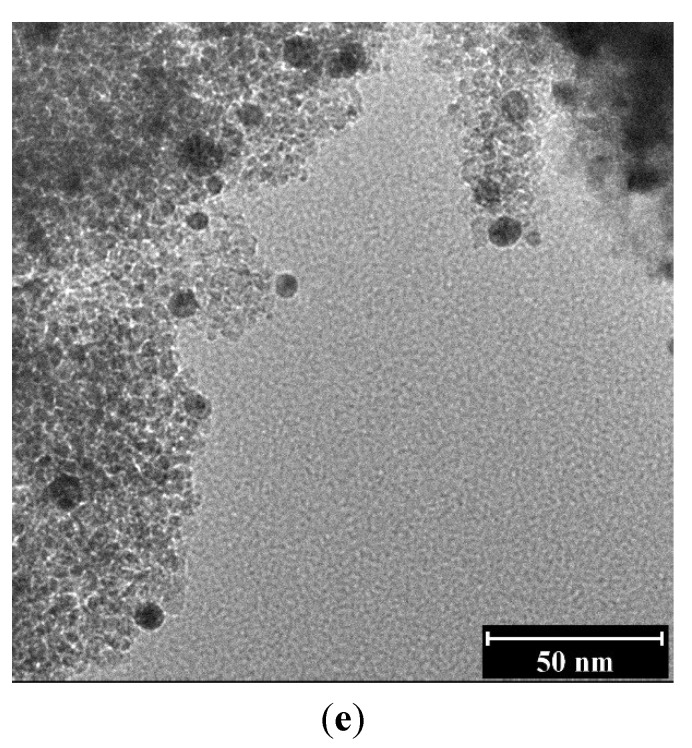
TEM images of (**a**) 5.00 × 10^−4^ M TC-stabilized Au sol (*D*_Au_ = 2.9 nm); (**b**) 1.25 × 10^−4^ M TC-stabilized Au-P25-CRIS (*D*_Au_ = 5.7 nm); (**c**) 5.00 × 10^−4^ M TC-stabilized Au-P25-CRIS (*D*_Au_ = 2.6 nm); (**d**) 0.63 × 10^−4^ M TC-stabilized Au-P25-CRSIM (*D*_Au_ = 6.7 nm); and (**e**) 2.50 × 10^−4^ M TC-stabilized Au-Kronos-CRIS (*D*_Au_ = 9.8 nm). Histograms representing the size distribution of Au were calculated according to all the TEM images taken of each sample.

The Au nanoparticles in the gold sol (Figure 3a) and also in the Aeroxide P25 CR samples (Figure 3b–d) were mainly spherical. The TEM image of a sample produced using Kronos Vlp7000 by the CRIS procedure revealed Au nanoparticles of about the same size as the TiO_2_ nanoparticles (Figure 3e; *D*_Au_ = 9.7 and *D*_TiO2_ = 7.8 nm).

**Figure 4:**
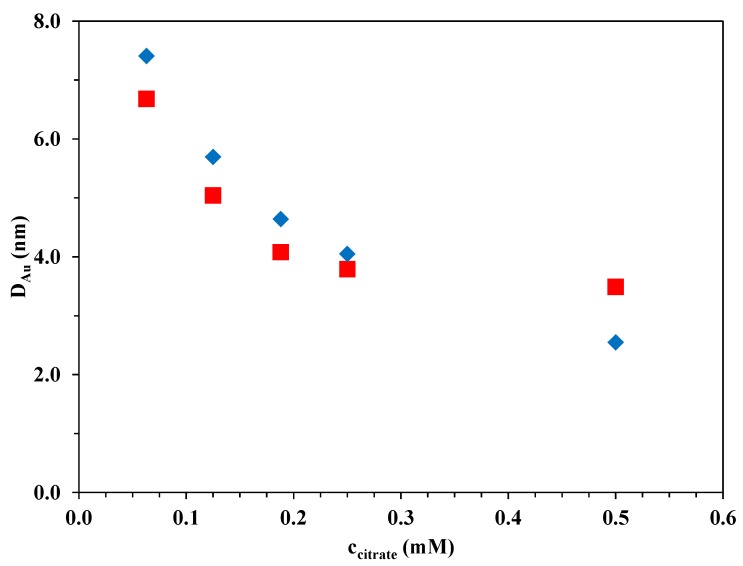
Dependence of the average Au nanoparticle size on the TC concentration during the synthesis (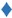 Au-P25-CRIS, 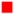 Au-P25-CRSIM).

The size distributions of the Au nanoparticles (~200 Au nanoparticles measured per sample) clearly demonstrated that the lower the stabilizing TC concentration during the chemical reduction, the larger the average size of the Au nanoparticles (Figure 4).

At low TC concentrations, the polydispersity of the Au nanoparticles was higher on both the CRSIM and the CRIS samples. The most homogeneous size distribution was achieved when the initial TC concentration was highest (5.00 × 10^−4^ M).

The photoreduced samples synthesized in the presence of citrate ions (PRTC) exhibited a much lower Au nanoparticle size as compared with the PROA catalysts, because citrate not only acts as an aiding electron donor in this reaction, but also as a stabilizing ion.

**Figure 5:**
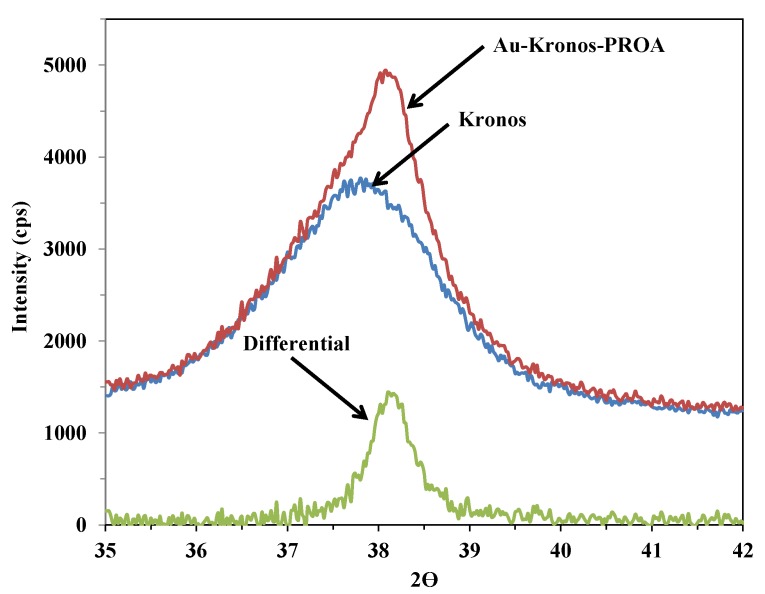
Differential XRD pattern of Au-Kronos-PROA and bare Kronos TiO_2_ photocatalysts.

Because of the small particle size of the Kronos Vlp7000 TiO_2_ photocatalyst, the Au and TiO_2_ nanoparticles could not be readily distinguished from each other. However, it was possible to determine the average Au particle size from the XRD peak of Au at 38.3° (2θ) (Figure 5) by using the Scherrer equation.

#### 3.1.3. Determination of Au Content with EDX

For selected samples, we examined whether the Au content deviated from the theoretical 1 wt%, but it emerged that the difference in catalytic activity was not caused by differing Au contents. Within statistical error, the Au loading of the prepared catalysts was as calculated. The measured sizes of the Au nanoparticles on the catalysts and the Au contents of the samples are presented in Table 1.

**Table 1:** Average Au particle diameters on the TiO_2_-based photocatalysts, and Au loading calculated from EDX measurements.

Sample	*D*_Au,XRD_ (nm)	*D*_Au,TEM_ (nm)	*c*_Au_ (wt%)
5.00 × 10^−4^ M citrate-CRSIM-P25	–	3.5	–
5.00 × 10^−4^ M citrate-CRIS-P25	–	2.6	–
2.50 × 10^−4^ M citrate-CRSIM-P25	–	3.8	–
2.50 × 10^−4^ M citrate-CRIS-P25	–	4.0	–
1.88 × 10^−4^ M citrate-CRSIM-P25	–	4.1	–
1.88 × 10^−4^ M citrate-CRIS-P25	–	4.6	–
1.25 × 10^−4^ M citrate-CRSIM-P25	–	5.0	~1.07
1.25 × 10^−4^ M citrate-CRIS-P25	–	5.7	~1.15
0.63 × 10^−4^ M citrate-CRSIM-P25	–	6.7	–
0.63 × 10^−4^ M citrate-CRIS-P25	–	7.4	–
PRTC-P25	–	18.8	–
PROA-P25	–	50.0	~1.01
2.50 × 10^−4^ M citrate-CRSIM-Kronos	5.9	–	–
2.50 × 10^−4^ M citrate-CRIS-Kronos	9.8	–	~0.99
1.88 × 10^−4^ M citrate-CRSIM-Kronos	5.8	–	~1.02
1.88 × 10^−4^ M citrate-CRIS-Kronos	6.2	–	–
1.25 × 10^−4^ M citrate-CRSIM-Kronos	7.0	–	–
1.25 × 10^−4^ M citrate-CRIS-Kronos	5.7	–	–
0.63 × 10^−4^ M citrate-CRSIM-Kronos	7.2	–	–
0.63 × 10^−4^ M citrate-CRIS-Kronos	6.4	–	–
PRTC-Kronos	10.4	–	–
PROA-Kronos	20.1	–	~0.99

XPS analysis of the samples revealed that despite the small size of the Au nanoparticles, gold is in metallic state Au^0^ on the surface. There was no indication of the presence of Au_2_O_3_ (Table 2).

**Table 2:** Oxidation states of the Au on the Au-TiO_2_ photocatalysts prepared by the CRIS method.

*D*_Au_ (nm)	Au^0^ 4f5/2 (87.82 eV)	Au^0^ 4f7/2 (84.15 eV)
7.4	45.59%	54.41%
5.7	44.94%	55.06%
4.0	46.12%	53.88%

### 3.2. Photocatalytic Measurements

#### 3.2.1. H_2_ Evolution from Oxalic Acid Solution

Under the present reaction conditions, we did not observe any H_2_ evolution from pure water. H_2_ production was measured in the presence of 50 mM oxalic acid in N_2_-purged suspensions. This high oxalic acid concentration was chosen to keep the substrate concentration decrease negligible: during the measurement, oxalic acid can decompose, mostly to CO_2_ and H_2_, under O_2_-free conditions. Furthermore, the initial concentration of oxalic acid, used as a sacrificial reagent for the H_2_ production measurements, was at least 100 times higher than the citrate concentration used during the syntheses. Most of the citrate ions were presumably eliminated during the washing procedure with oxalic acid and water.

**Figure 6:**
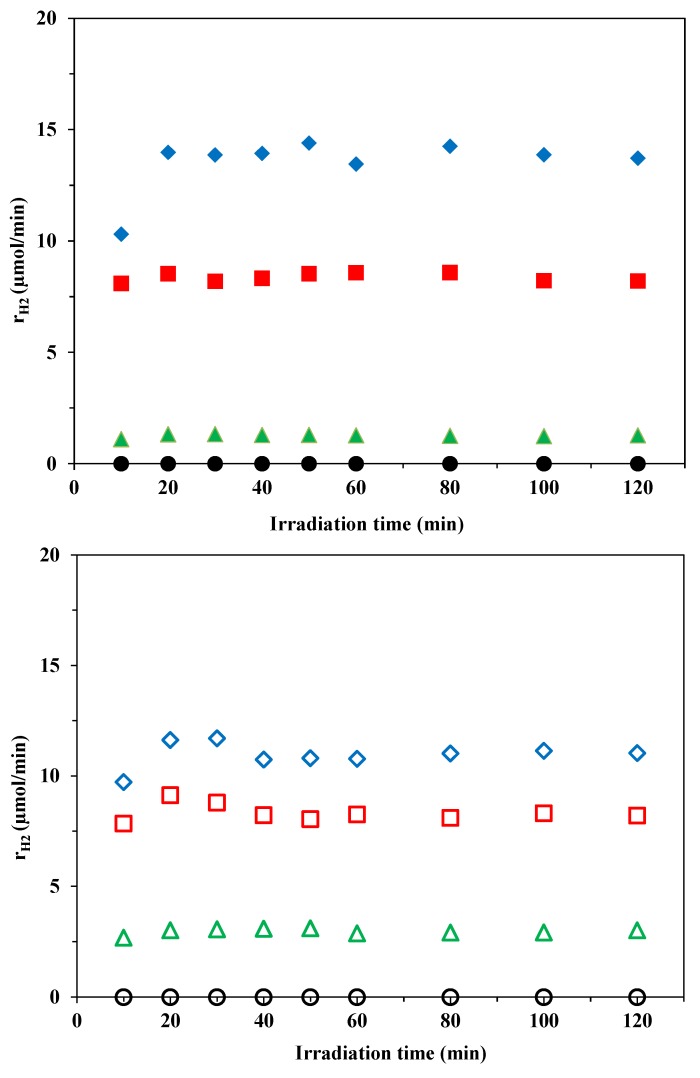
Comparison of H_2_ evolution rates as a function of irradiation time on bare TiO_2_, PR Au-TiO_2_ and the best-performing CR Au-TiO_2_ catalysts (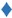 Au-P25-CRIS_opt_, 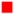 Au-P25-CRSIM_opt_, 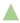 Au-P25-PROA, ● P25; 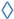 Au-Kronos-CRIS_opt_, 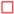 Au-Kronos-CRSIM_opt_, 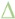 Au-Kronos-PROA, ○ Kronos).

Au-doped TiO_2_ catalysts exhibited almost stable H_2_ evolution, whereas there was a huge decrease in H_2_ production in the first 40 min of our earlier experiments with Pt-TiO_2_ [19]. For all the Au-modified TiO_2_ catalysts, the H_2_ production curves reached a saturation level in the first 20 min and the H_2_ evolution rate subsequently remained nearly constant for the remainder of the experiment (Figure 6). The H_2_ evolution rate is strongly influenced by the size of the deposited Au particles. Under these conditions, the bare TiO_2_ (pure Aeroxide P25 or Kronos Vlp7000) displayed only slight photocatalytic activity for H_2_ production. However, the chemically reduced Au-deposited titanias with optimum size distribution of the Au on the surface manifested 11-fold (Au-P25) or 4-fold (Au-Kronos) higher photocatalytic activity than that of the respective photoreduced sample. The CRIS samples were significantly more efficient than those made by the CRSIM method. This can most probably be explained by the better distribution of the Au nanoparticles on the TiO_2_ surface in the case of the CRIS samples.

**Figure 7:**
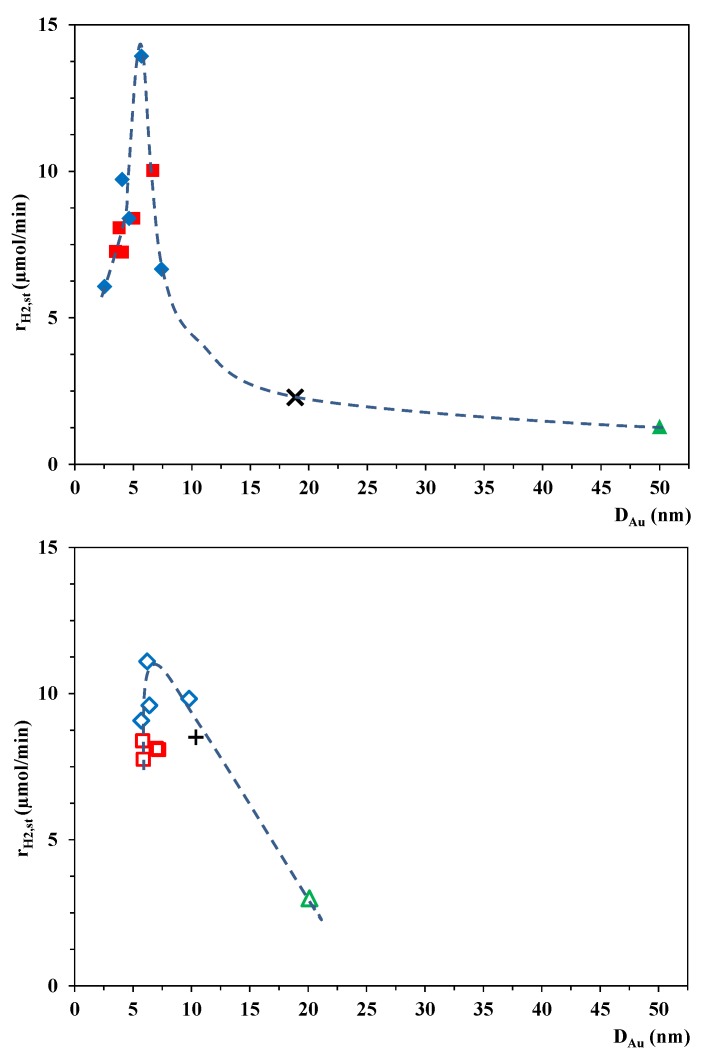
Average H_2_ evolution rates as a function of average Au particle size on different Au-modified TiO_2_ photocatalysts: (**a**) Aeroxide P25 TiO_2_-based samples (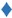 Au-P25-CRIS, 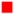 Au-P25-CRSIM, 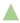 Au-P25-PROA, **×** Au-P25-PRTC); (**b**) Kronos Vlp7000 TiO_2_-based samples (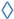 Au-Kronos-CRIS, 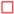 Au-Kronos-CRSIM, 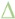 Au-Kronos-PROA, **+** Au-Kronos-PRTC).

In view of the steady-state overall rates of H_2_ production, we compared these values with the average Au particle size observed on each catalyst. Two parallel samples were synthesized and tested photocatalytically for all investigated Au-TiO_2_ samples, with good reproducibility (within ±5%). Figure 7 presents the connection between the Au particle size on the TiO_2_ and the H_2_ production rate. Larger Au nanoparticles (*D*_Au_ > 10 nm) resulted in clearly lower photocatalytic activities. The catalysts that were synthesized by the CR method performed much better in producing H_2_ from oxalic acid solution than those made by the PR method, probably because of the much larger Au particles on the PROA TiO_2_ samples. However, at lower Au particle sizes, there was a maximum in the photocatalytic activity at *D*_Au_ ≈ 6 nm. Smaller Au nanoparticles are unfavorable, probably because of the loss in metallic character [45,46]. It was concluded that the most important parameter influencing the H_2_ production efficiency of Au-P25 photocatalysts appears to be the Au nanoparticle size. The optimum size of the Au nanoparticles with the best distribution on the TiO_2_ surface was achieved with the CRIS method.

**Figure 8:**
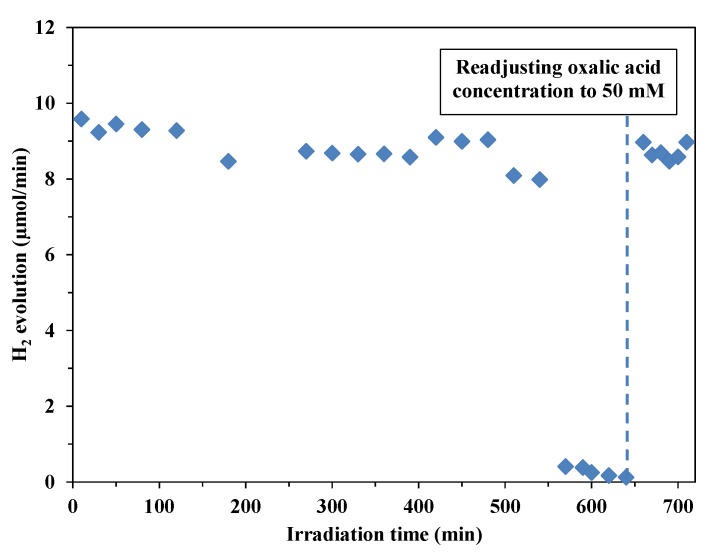
Long-term irradiation and H_2_ evolution rates on Au-TiO_2_ photocatalyst in a suspension containing oxalic acid (*c*_OA, initial_ = 50 mM).

To examine the long-term usability of this catalyst, Au-P25 (CRSIM_opt_, *D*_Au_ = 6.7 nm) suspension was irradiated until the OA had been fully mineralized (*c*_OA, initial_ = 50 mM). The lamps were then turned off for 10 min and the OA concentration was readjusted to 50 mM through mixing of the required amount of oxalic acid powder into the suspension. After the UV irradiation had been turned on again, the H_2_ evolution rate was restored to the same level as at the beginning of the experiment (Figure 8).

It can be concluded that these catalysts will not lose their catalytic activity as long as an organic sacrificial agent is present in the suspension. Through a continuous supply of sacrificial reagent, a nearly constant photocatalytic H_2_ evolution rate can be achieved.

#### 3.2.2. Decomposition of Oxalic Acid under Anaerobic Conditions

**Figure 9:**
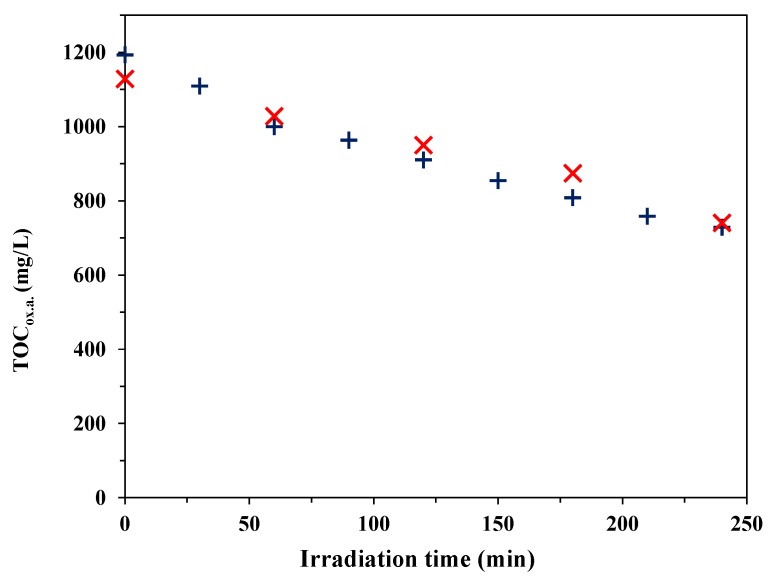
Photocatalytic decomposition and mineralization of oxalic acid under UV irradiation and anaerobic conditions with the Au-P25-CRIS (*D*_Au_ = 5.7 nm) photocatalyst (× HPLC measurement, + TOC measurement).

We investigated the correlation between the H_2_ production and the diminution of oxalic acid under the same conditions. The residual OA concentration was determined by HPLC and TOC analysis (Figure 9). For this experiment, we used the Au-doped photocatalyst that performed best in the H_2_ production measurements. The average rate of H_2_ evolution on this catalyst was 1.546 × 10^−6^ mol/Ls, while the average rate of oxalic acid decomposition was 1.345 × 10^−6^ mol/Ls. The actinometric measurement for the reactor (*I* = 0.954 × 10^−5^ mol photon/s) indicated that the apparent quantum yield was 4.86% for H_2_ production and 4.22% for oxalic acid decomposition, via the following reaction (Equation (1)):

(COOH)_2_ + 2 h^+^ + 2 e^−^ = 2 CO_2_ + H_2_ (1)

When our results are compared with the available apparent quantum efficiency of Au-TiO_2_ data in the literature [47,48], the present photocatalyst with size-optimized gold nanoparticles is very promising.

#### 3.2.3. H_2_ Production from Methanol

**Figure 10:**
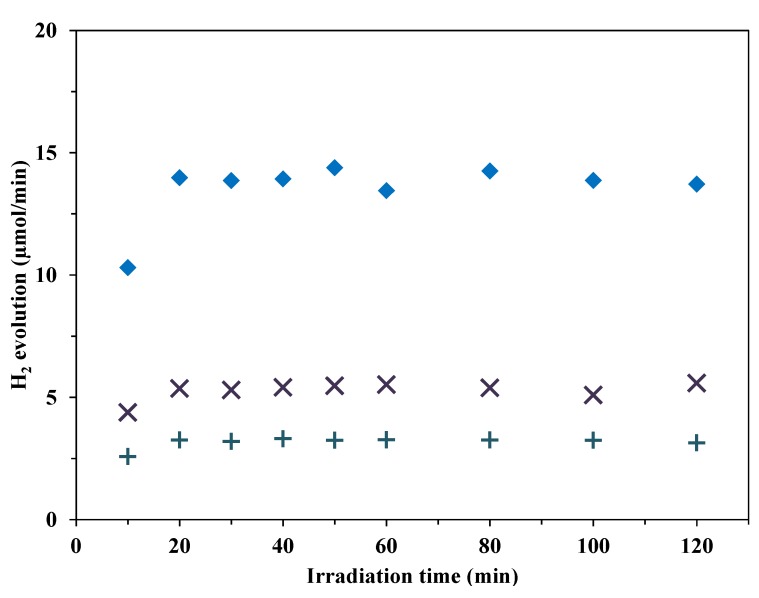
H_2_ evolution from 50 mM oxalic acid, 50 mM and 100 mM methanol (initial concentrations), using Au-loaded P25-CRIS photocatalyst, *D*_Au_ = 5.7 nm (♦ 50 mM oxalic acid, + 50 mM methanol, × 100 mM methanol).

Methanol is a widely used sacrificial agent in photocatalytic H_2_ production measurements. We examined the photocatalytic activity of our best-performing Au-deposited P25-based catalyst (CRIS sample, *D*_Au_ = 5.7 nm) in H_2_ evolution when the suspension contained methanol instead of oxalic acid as a readily oxidizable organic component. In one case the initial concentration of methanol (50 mM) and in another experiment the initial carbon content (TOC = 1200 ppm, *c*_methanol_ = 100 mM) were the same as in the measurements with oxalic acid. Methanol resulted in a much lower rate of H_2_ evolution than that with oxalic acid under the same reaction conditions (Figure 10). The maximum apparent photonic efficiency was measured as 1.13% with 50 mM and 1.89% with 100 mM methanol, and 4.86% with 50 mM oxalic acid. This means that chemisorbed oxalic acid is a more efficient electron donor (hole scavenger) than methanol on the use of Au-TiO_2_.

### 3.3. Adsorption of Oxalic Acid on Bare TiO_2_ Catalysts

**Figure 11:**
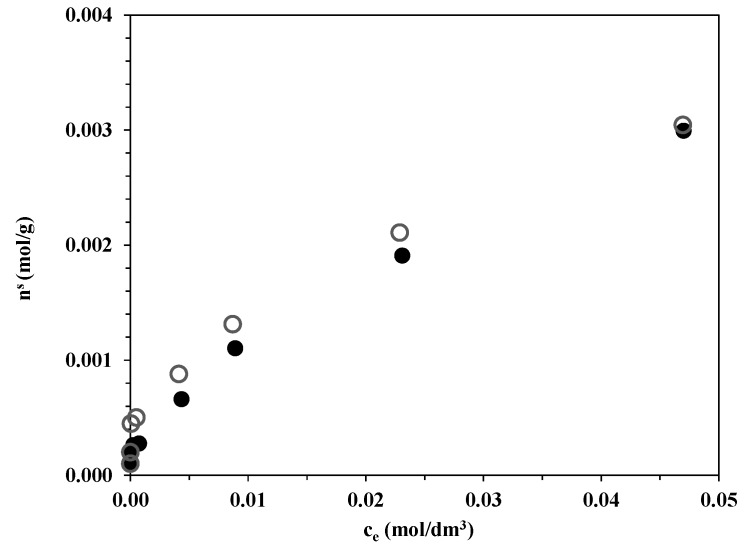
Adsorption isotherms of OA on ● bare Aeroxide P25 and ○ bare Kronos Vlp7000 TiO_2_ photocatalysts at 25.0 °C.

The H_2_ production measurements revealed a surprisingly small difference between the H_2_ evolution rates on Aeroxide P25-based and the Kronos TiO_2_ catalysts with similar Au particle sizes, in contrast with the significant difference in their specific surface areas. The specific surface area of bare Aeroxide P25 TiO_2_ was 49.0 m^2^/g, while that of Kronos Vlp7000 TiO_2_ was 296.5 m^2^/g. One weight percent Au deposition on our catalysts did not affect the BET specific surface area considerably. The adsorption properties of oxalic acid were therefore investigated on these two bare catalysts in 1 g/L TiO_2_ suspensions kept at 25 °C in the dark for 4 h. Samples were then taken from the supernatant and the residual TiO_2_ particles were filtered. The HPLC measurements indicated that Kronos Vlp7000 adsorbed only slightly more oxalic acid on its surface than did Aeroxide P25 (Figure 11). At the initial oxalic acid concentration applied in the H_2_ production experiments (50 mM), all the binding sites of the TiO_2_ catalysts were likely to be covered by oxalate ions. The UV oxalic acid decomposition experiment under O_2_-free conditions indicated that the concentration did not decrease below 30 mM during the 2 h reaction time; at this concentration, there was still only a minor difference in the oxalic acid adsorption capacity between the two bare photocatalysts.

BET surface area measurements on the Au-loaded TiO_2_ samples demonstrated that 1 wt% Au had only a slight impact on the specific surface area (Au-P25: 48.7 m^2^/g, Au-Kronos: 266.3 m^2^/g), which indicates that the Au deposition cannot be responsible for the similar amounts of oxalic acid adsorbed.

## 4. Conclusions

Differently sized Au nanoparticles were synthetized on two kinds of TiO_2_ (Aeroxide P25 and Kronos Vlp7000), either by chemical reduction or by photoreduction, with constant Au content (1 wt%). The size of the Au nanoparticles could be finely regulated (especially in the range of 2–10 nm) through the use of different concentrations of the stabilizing agent. Two chemical reduction methods (CRIS and CRSIM) were utilized and the size distribution and monodispersity of the Au particles were also investigated. In UV-irradiated O_2_-free suspensions, the Au-modified TiO_2_ catalysts exhibited much higher H_2_ production activities, while the bare catalysts displayed insignificant H_2_ evolution in the presence of oxalic acid. The photocatalytic activity proved to depend strongly on the average Au particle diameter: there was a H_2_ production rate maximum at *D*_Au_ = 5.7 nm for Aeroxide P25, and at *D*_Au_ = 6.2 nm for Kronos Vlp7000. The highest rate of H_2_ production was achieved with the samples prepared by the CRIS method, which provided the most homogeneous distribution of the Au nanoparticles on the TiO_2_ surface.

Although the surface areas of these two commercially available TiO_2_ catalysts differ significantly, the rate of H_2_ evolution from oxalic acid was more or less the same when Au nanoparticles of almost the same average size were present on their surfaces. The best-performing photocatalyst demonstrated much higher H_2_ production activity when oxalic acid was used as sacrificial reagent rather than the widely used methanol. The constant H_2_ evolution rates attained during the experiments would allow the use of these catalysts over a long irradiation period without significant loss in photocatalytic activity.
